# Factors Influencing Public Participation in Community Disaster Mitigation Activities: A Comparison of Model and Nonmodel Disaster Mitigation Communities

**DOI:** 10.3390/ijerph191912278

**Published:** 2022-09-27

**Authors:** Ting Que, Yuxin Wu, Shiyu Hu, Jianmin Cai, Nan Jiang, Huige Xing

**Affiliations:** 1 College of Architecture and Environment, Sichuan University, Chengdu 610065, China; 2 State Key Laboratory of Hydraulics and Mountain River Engineering, Sichuan University, Chengdu 610065, China

**Keywords:** disaster risk, disaster prevention, public participation, community resilience, disaster mitigation community building

## Abstract

Public participation in community-organized disaster mitigation activities is important for improving disaster mitigation capacity. With data from 260 questionnaires, this study compared the current status of public participation in model disaster mitigation communities and nonmodel communities in a geological-disaster-prone area. Three community-organized disaster mitigation education activities were compared cross-sectionally. A binary logistic regression was used to analyze the effects of attitude, perceived behavioral control, disaster experience, and other key factors on the public’s choice to participate in community disaster mitigation activities. The analysis results indicated that model communities had higher public participation in two efforts, evacuation drills and self-help skills training, and lower participation in activities that invited them to express their feedback than nonmodel communities. The influence of attitudinal factors on the decision to participate in disaster mitigation activities had a high similarity across community types. The public participation in model disaster mitigation communities is influenced by factors such as subjective norms and participation cognition; the behavior of people in nonmodel communities is influenced by factors such as previous experience with disasters, perceived behavioral control, risk perception, and participation cognition and has a greater potential for disaster mitigation community construction. This study provides practical evidence and theoretical support for strengthening the sustainable development of disaster mitigation community building.

## 1. Introduction

The geological environment in Southwest China is complex and susceptible to extreme weather conditions. Coupled with the impact of frequent human engineering activities, geological disasters such as landslides and mudslides have occurred repeatedly, most notably in the Wenchuan earthquake in 2008, which induced landslide disasters in up to 200,000 places [1]. Approximately 80% of Sichuan Province is located in mountainous areas, and geological disasters in these areas seriously threaten the safety of people’s lives and property. To protect people’s rights and interests and strive to improve the regional capacity for sustainable disaster mitigation, the Chinese government has increased its investment in disaster prevention and mitigation construction, including USD 48.68 billion in national spending on “disaster prevention and emergency management” for 2019–2020. The disaster mitigation conference held in 2018 pointed out that it is necessary to improve the leadership and emergency response capabilities of grassroots communities in disaster mitigation work and to expand the role of comprehensive disaster mitigation model counties and demonstration communities [2]. The “Decision of the Central Committee of the Communist Party of China on Several Major Issues Concerning Comprehensively Deepening the Reform” proposes a transition from national disaster relief to national disaster mitigation and from a government-led approach to a new pattern of government-advised social coordination and public participation.

In the study of public participation in disaster prevention and mitigation, Pearce argues that public participation combined with community governance can contribute to achieving sustainable disaster mitigation goals [3]. The bottom-up disaster prevention and mitigation process helps refine the disaster mitigation strategy system, enhance people’s understanding of disaster risks, improve overall disaster prevention and mitigation capabilities, and reduce disaster losses. With the promotion of the importance of public power in disaster mitigation, how to promote public participation in community disaster risk management has become a current research hotspot, and individual risk perception [4], willing preparedness [5], and public participation in community disaster mitigation models [6] have received increasing attention from research scholars. Disaster mitigation activities are an important way to promote public participation. On the one hand, communities provide the opportunity for the public to participate in disaster mitigation education and community governance by organizing evacuation drills, emergency self-rescue skills, and other training activities; on the other hand, in the process of participating in disaster risk management [7,8], individual risk awareness and coping ability is strengthened through active learning and training [9]. This two-way interaction and communication contribute to the improvement of community disaster risk reduction capabilities.

Since 2008, the Chinese government has awarded the title of “National Comprehensive Disaster Reduction Demonstration Community” to communities that meet the requirements of the “Management Measures for the Creation of National Comprehensive Disaster Reduction Demonstration Communities” and pass the audit. China’s comprehensive disaster reduction demonstration communities (hereafter referred to as “model communities”) focus on strengthening the construction of community shelters, stockpiling materials, and enhancing the awareness and capacity of community residents in disaster reduction [10]. Wu et al. [11] found that model communities have significant disaster reduction benefits and that the continued construction of a model community in one area can effectively lead to disaster reduction in surrounding communities. “Disaster risk reduction communities” emphasize the motivation of individuals to participate in disaster prevention and mitigation activities under the leadership of the government and encourage all residents to join the community disaster prevention and mitigation team. The practice of training disaster-reducing communities in the United States proves that resilient disaster-reducing communities can recover more quickly in the post-disaster reconstruction process [12]. Cases from six developing countries in the Caribbean islands illustrate that participatory disaster management approaches can provide local knowledge of policy development while providing the public with access to resources [13]. The community-centered disaster mitigation strategy is in line with China’s national conditions and will play an important role in disaster management. China has so far formed a preliminary community disaster management system, relevant regulations have been promulgated and implemented [14], and a disaster prevention and mitigation system with Chinese characteristics oriented to the national conditions of China has been established [15].

Research on the construction of disaster mitigation communities has focused on the evolution of community space, macro-policies, and the qualitative evaluation of disaster mitigation capacity [16,17], but with the advancement of participatory disaster management, many scholars believe that disaster risk reduction strategies need to promote active community public participation [18]. Research in this area mainly focuses on a specific disaster mitigation activity, such as evacuation drills, but a single activity has limited ability to improve disaster mitigation capacity. Especially for remote rural areas in disaster-prone areas, due to the current situation of lower economic resources and varying levels of literacy, it is even more necessary to diversify disaster mitigation activities to meet the public’s preferences for participation. Diversification of disaster mitigation activities is important to improve the initiative and enthusiasm for public participation and to promote community disaster prevention and mitigation. Therefore, it is necessary to conduct a cross-sectional comparison of the factors influencing the public’s decisions to participate in different disaster mitigation activities, especially those that are organized locally to help communities make flexible adjustments to disaster mitigation programs according to their current capacities and public preferences.

In this paper, we used model disaster mitigation communities and nonmodel disaster mitigation communities in Jinchuan County, a geohazard-prone area, to distribute questionnaires and conduct semistructured interviews to investigate the factors influencing public participation in “evacuation drills”, “self-help skills training”, and “opinion expression”. The main objectives of this study are as follows: (1) to investigate the degree of public participation in different disaster mitigation activities in both model and nonmodel disaster mitigation communities, (2) to identify the specific factors influencing decisions to participate publicly in different disaster mitigation activities in different community types and analyze the influence of these factors on public participation, and (3) to provide reasonable countermeasures and suggestions for different communities to improve public enthusiasm for participation and enhance the construction of disaster mitigation communities according to the influencing factors on participation in different disaster mitigation activities.

## 2. Theoretical Basis

### 2.1. Community-Based Disaster Risk Management

The community-based disaster risk management approach has attracted widespread attention from scholars since 2000, aiming to reduce community vulnerability, formulate disaster mitigation strategies and interventions based on local community characteristics and indigenous knowledge, and improve community resilience and recovery when faced with disaster threats [19]. This approach has proven to be sufficiently adaptive during disaster preparedness and reconstruction periods [20]. Resilient communities can make use of limited inputs to overcome the difficulties caused by a lack of resources even with a large number of potentially concealed disaster sites. In Wanzhou District, Three Gorges Reservoir Area, China, a disaster risk management system based on disaster mitigation communities has improved community resilience through a simple yet efficient monitoring system and the implementation of long-term risk education [21]. Wang stated that combining scientific knowledge with indigenous knowledge is an important means to improve community capacity for reducing disaster risk [22]. In the earthquake and tsunami disaster in Chile in 2010, participation, trust, cooperation, and knowledge within the disaster mitigation community played a crucial role in the community’s response to the disaster [23]. Community-based disaster resilience assessment [24], community resilience assessment measures [25,26], investment in participatory early warning systems [27], etc., have also become hot issues. Burton used the case of Hurricane Katrina to validate community resilience indicators for natural hazards and disasters, providing a valid scientific basis for the resilience evaluation of disaster mitigation communities.

Actively implementing disaster risk reduction activities is an important approach in risk education and community preparedness. Witvorapong et al. [28] found through an empirical survey that members of the public who actively participate in community disaster mitigation activities are 5.2% more likely to take risk reduction actions than those who never participate, indicating that extensive disaster risk education actions are feasible and necessary. At present, common disaster mitigation activities mainly include evacuation drills, first-aid skills training, and the solicitation of feedback. Evacuation drills are regarded as the most important community activities in the context of disaster risk reduction. This activity can strengthen the public’s emergency survival ability by shortening evacuation times or clarifying escape routes [8]. People with experience are more likely to be evacuated successfully when a disaster occurs [29]. Community-organized first-aid skills training educates residents about first-aid knowledge and skills [30,31], which is an important mitigation measure to reduce human casualties in a disaster. The solicitation of feedback provides an opportunity for two-way communication between the public and decision-makers so that they can proactively discover and solve problems in local governance [32,33].

### 2.2. Factors Affecting Public Participation in Disaster Mitigation Activities

In terms of public participation in disaster mitigation activities, most studies have concluded that factors such as risk perception [34,35], disaster experience [36], knowledge of participation mechanisms [37], and information access [38] encourage individuals to adopt mitigation measures. Risk perception is considered an assessment of the perceived probability (likelihood) and perceived consequences (severity) of a hazard [39]. A survey on flood risk perception found that risk perception is an important factor that motivates individuals to take preventive measures and support policies aimed at reducing flood risk [40]. Studies are increasingly exploring the relationship between risk perception and mitigation behavior, especially when individuals recall past damage or imagine the impact of disasters and consider the influence of factors such as trust in government and response assessment [41,42]. Research in the field of disaster response often uses past experiences as predictors of the dependent variable [43]. Disaster experience not only enhances individual understanding of actual risk and willingness to mitigate disaster risk but also motivates affected communities to pay attention to the implementation of disaster reduction measures. Hoffmann and Muttarak [44], through a survey in the Philippines and Thailand, found that the propensity to share experiences and take effective joint actions to prepare for future disasters may be stronger if multiple households in a region are affected by a disaster. The public requires a level of cognition of participatory activities, including an understanding of disaster mitigation activities and attention to participation pathways [45]. Wu’s survey of 3245 members of the public in 31 provinces of mainland China who adopted earthquake preparedness activities found that concern for risk reduction measures and participation in public affairs were positive predictors of physical and conscious preparedness [46]. Lee [47] discussed the obstacles Nepal has encountered in disaster management, for which a lack of community-level engagement and understanding is an important factor hindering the improvement of disaster resilience and coping capabilities.

In the study of individual behavioral decision-making, factors such as individual psychology and social relationships should be considered [48]. The theory of planned behavior [49] is derived from the theory of reasoned behavior (TRA) [50], which is widely used to predict or explain behavioral intentions or actual behaviors [51,52]. The theory of planned behavior suggests that an individual’s intention to adopt behavior is influenced by three main factors: attitude, subjective norm, and perceived behavioral control. Among these, attitude refers to the perceived value of the behavior, whether positive or negative; subjective norm refers to social pressure from influential people or surrounding groups [53]; and perceived behavioral control involves assessing the likelihood of performing the behavior with the available resources and opportunities [49]. Ajzen [54] argues that perceived behavioral control consists of two components: self-efficacy, which emphasizes the evaluation of one’s own ability to carry out a plan, and locus of control, which indicates the intensity of feelings of personal control over extrinsic factors. The theory of planned behavior has been used in several attempts to explain preparedness behavior in natural disasters, such as earthquakes [55], typhoons [56], climate change [57], floods [58], and other phenomena. Zaremohzzabie’s [59] study of household earthquake behavior in Malaysia found that preparedness attitudes, subjective norms, community involvement, and community institutional trust were important predictors of behavioral intentions to engage in earthquake preparedness. Luo [60] argued that the adoption of mitigation behaviors by farmers depends on their attitudes toward multiple risk measures and the cost of mitigating disasters.

**Figure 1:**
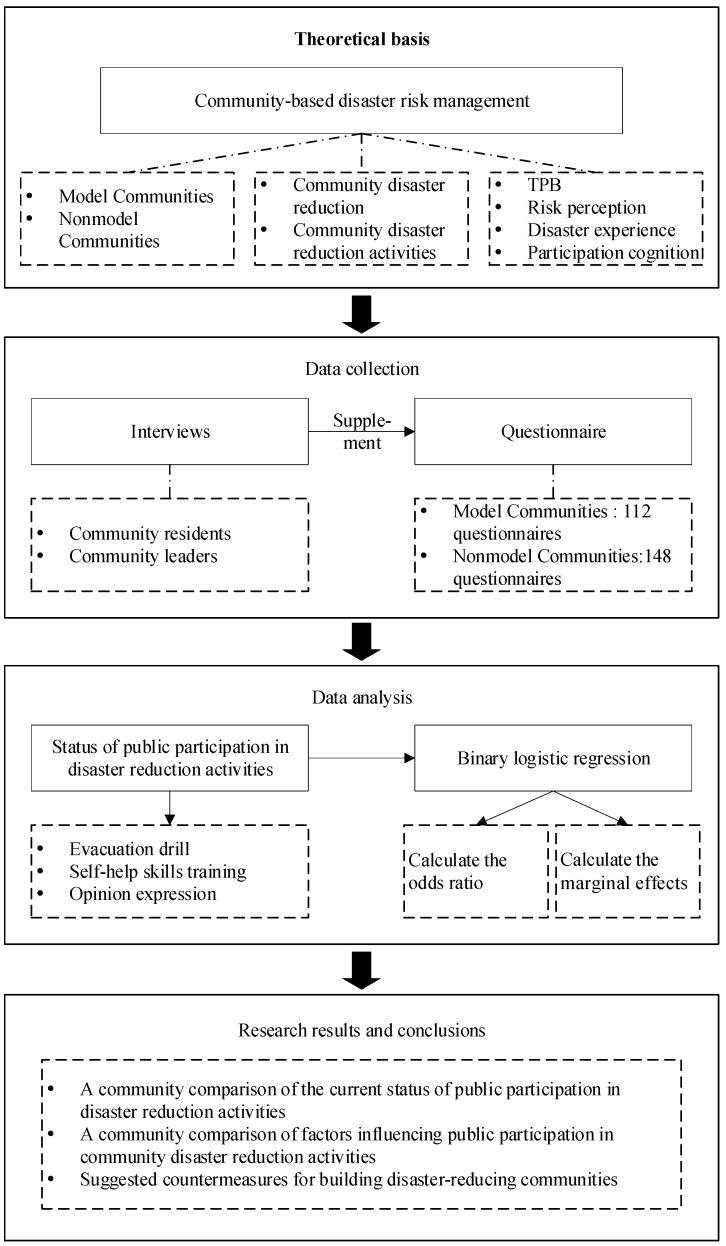
Technology road-mapping. The data for the model communities are from the Mulin community, and the data for the nonmodel communities are from Desheng Village, Sha’er Township, Shangengzi Village, and Danzhamu Village.

To summarize, public participation in diverse community disaster mitigation activities is an important task of disaster risk management at present. Based on previous research, this paper comprehensively selects attitudes, subjective norms, perceived behavior control, risk perception, disaster experience, and participation cognition from the perspectives of psychology, natural environment, and social relations to investigate the influencing factors on public behavior in various disaster mitigation activities. To provide an empirical basis for the targeted improvement of community disaster mitigation plans, a comparative analysis was conducted with public participation in model and nonmodel communities. Figure 1 depicts the technical path of this study.

## 3. Materials and Methods

### 3.1. Study Area

**Figure 2:**
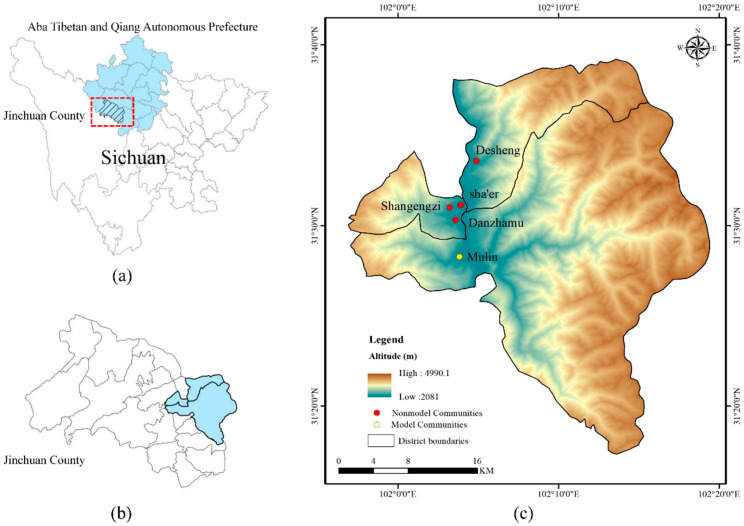
Site location of the study area. (**a**) Administrative map of Sichuan Province. (**b**) Jinchuan County administrative map and survey of townships. (**c**) Distribution of field survey sites.

Jinchuan County is located in the northwestern part of Sichuan Province and the southwestern part of the Aba Tibetan and Qiang Autonomous Prefecture in the transition zone between the Sichuan Basin and the southeastern edge of the Qinghai–Tibet Plateau (as shown in Figure 2). Influenced by the continental plateau monsoon climate, it is extremely susceptible to geological hazards such as mudslides during the peak rainfall period of June to September. One study found that a total of 256 threatening mudslide ditches in Jinchuan County could be identified by GF-1 satellite images, including 6 large, 161 medium, and 89 small ditches [61]. To reduce the risks of geological hazards and protect people’s lives and property, the Jinchuan County government has taken many disaster management measures, especially the mobilization of public participation in disaster mitigation, which has achieved remarkable results. For example, in “the 6.27 Zengdagou mudslide”, villagers reported to the higher authorities in time to sound the geological disaster warning and evacuate and transfer people out of the danger zone successfully in advance.

### 3.2. Sample Collection and Interviews

The model community was selected from the Mulin community in Lewu Township, Jinchuan County, which is the only community in Jinchuan County to be named a “National Comprehensive Disaster Reduction Model Community for 2020”. The sample of nonmodel communities was selected from villages that have experienced flash floods and mudslides in the past and are vulnerable to secondary hazards, including Desheng Village, Sha’er Township, Shangengzi Village, and Danzhamu Village. The geographical location of the sample collection sites is shown in Figure 2c. One person from each household was selected to fill out a questionnaire survey, and residents who were unwilling to participate in the survey and those who did not understand the subject matter of the questionnaire were excluded. A total of 300 questionnaires were distributed, and 260 were valid, with a return rate of 86.7%, including 112 in model communities and 148 in nonmodel communities.

After the questionnaire was completed, survey team members invited participants to an interview for broader information on community-based disaster risk reduction. Participants who agreed to participate in the interview could respond to the interview questions as they saw fit, and the survey team members were responsible for taking notes. Participants first identified themselves as “community residents” or “community leaders” and volunteered to answer three questions: (1) How do you conduct disaster risk reduction education activities in your community? (2) How do you think participating in community-organized disaster risk reduction education activities has helped you personally? (3) Do you have any suggestions for mobilizing residents to participate in community-based disaster risk reduction activities? A total of 25 interviewees were recruited for the interviews, including 14 from model communities and 11 from nonmodel communities.

### 3.3. Measurement Tools

The questionnaire consisted of three parts. The first part introduced the purpose and background of the study and the basic demographic characteristics. In the second part, the dependent variables were measured, and the three dependent variables of “evacuation drill”, “self-help skills training”, and “opinion expression” were answered as single-choice questions addressing “whether to participate”. The third section of the questionnaire was the core section, which assessed independent variables such as attitude [49,62], subjective norms [49,63], perceived behavioral control [49,64], risk perception [65], disaster experience [66], and participative cognition [63,67]. All independent variables were measured using multiple indicators, mostly adapted from previous literature to suit the current research setting. All independent variables were measured using a five-point Likert scale, and all items were described positively.

### 3.4. Data Processing Methods

The previous methods of questionnaire analysis include linear regression, logistic regression, structural equation modeling, etc. Since the dependent variable is an independent binary variable, this study processed the data using binary logistic regression to examine the relationship between the independent factors and event likelihood [68]. The odds ratio (OR) and marginal effects were calculated when the community chose different participation activities, and the specific factors affecting public participation were analyzed and predicted. The OR describes the direction of the likelihood that the public will choose to engage in disaster mitigation activities when one of the predictors increases by one unit while the other variables remain the same [69]. If the OR is greater than 1, it indicates that the increase in this predictor is positively correlated with an increase in the chance of public decisions to act, and if the OR is less than 1, the situation is the opposite [70]. The OR can reveal the direction of the relationship but does not reflect the magnitude of the probability. Therefore, this paper further predicts the effect of independent variables on public participation by calculating the mean marginal effect result from logistic regression. Marginal effects are partial derivatives or instantaneous rates of change for continuous independent variables and discrete rates of change for categorical independent variables. The mean marginal effect results directly demonstrate the likelihood that a change in the predictor variable leads to a change in the dependent variable, with other variables held constant at their mean values.

The logistic regression model takes the following form:

(1) $$\log\left[\frac{p_{i}}{1-p_{i}}\right]\;=\;\alpha +\beta_{1}x_{1}+\beta_{2}x_{2}+\ldots +\beta_{k}x_{k}$$

The OR can be expressed as follows:

(2) $$\mathrm{Odds}\;\mathrm{ratio}=\;\mathrm{Exp}\left(\beta \right)\;=\;\frac{p\left(x+1\right)/\left(1-p\left(x+1\right)\right)}{p\left(x\right)/p\left(1-p\left(x\right)\right)}$$

The marginal effect can be expressed as follows:

(3) $$\mathrm{ME}\;=\;\frac{\Delta y}{\Delta x}$$

where *i* denotes an individual; $p_{i}$ denotes the probability of the event occurring; $1-p_{i}$ denotes the probability of the event not occurring; α denotes the intercept; β denotes the regression coefficient; and *x* denotes the independent variable [71,72,73].

## 4. Results

### 4.1. Demographic Characteristics of the Sample

The statistical results of the sample demographic variables are shown in Table 1, and the main characteristics are as follows:

(1) There were more women than men in the sample size;
(2) The age level of the respondents was concentrated in the 46–60 age group;
(3) The educational level of respondents in nonmodel communities was significantly lower than that in model communities;
(4) In terms of occupation, most respondents in the nonmodel communities were farmers, while respondents in the model communities were mainly farmers and self-employed individuals;
(5) The monthly income of respondents in nonmodel communities was generally low, with 65.54% earning less than RMB 1500 per month, while 47.33% of respondents in model communities earn more than RMB 3000 per month.

Overall, the range of social groups covered by the respondents in this survey and the sample information were highly representative and conducive to the follow-up study.

### 4.2. Status of Public Participation in Disaster Mitigation Activities

**Figure 3:**
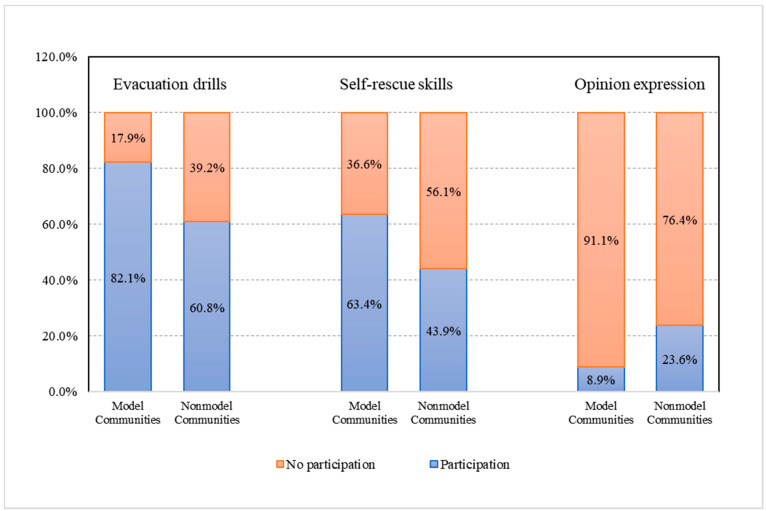
Percentage of public participation in disaster mitigation activities in model and nonmodel communities (%).

As shown in Figure 3, the proportion of community participation in disaster mitigation activities shows that there are significant differences in the public’s choices to participate in different disaster mitigation activities by community type. The results indicated the following: (1) community respondents have a high degree of participation in “evacuation drill” activities, with 82.1% of model communities participating in this activity and 60.8% of nonmodel communities participating in this activity; (2) in total, 63.4% of the respondents from model communities participated in the “self-help skills training” activity, and 43.9% of the respondents from nonmodel communities participated in the activity, with the former being significantly higher than the latter; (3) the results of public participation in the “opinion expression” activity are special, as most of the public did not participate in either community, with 8.9% participating in model communities and 23.6% participating in nonmodel communities, the former being slightly lower than the latter.

### 4.3. Factor Dimensionality Reduction

The factors with the same characteristics were normalized and simplified through dimensionality reduction processing to preserve the integrity of the information to the maximum extent and provide optimized results for the subsequent analysis [74]. First, the Kaiser–Meyer–Olkin (KMO) test value of the scale was 0.780 (generally required to be greater than 0.6), and Bartlett’s spherical test value was 2100.573, *p* < 0.001, indicating that the collected data were suitable for factor analysis. Principal component analysis and orthogonal rotation were used to extract the common factors with eigenvalues greater than one. The results are shown in Table 2. Six main factors are extracted from this scale, factor-related questions are consistent with the expected variables, and the cumulative variance contribution rate of factor extraction reaches 67.96% (more than 60%), which indicates that the measurement scale has good validity.

### 4.4. Different Community Types Impact Public Participation in Disaster Mitigation

To investigate the factors influencing public choices to participate in community disaster mitigation activities in different community types, a *t*-test was needed to examine the variability in the level of variables between the two types of communities. Table 3 shows the results of descriptive statistics and *t*-tests for the independent variables between the two communities. The results show that there was a significant difference (Sig < 0.05) in the mean values of each variable between different community types.

The mean values of “attitude”, “subjective norms”, and “participation cognition” in the model communities were significantly higher than those in the nonmodel communities. This indicated that the model communities have positive attitudes and higher awareness of participatory disaster mitigation activities and are more likely to be influenced by the community and have a stronger sense of consensus.

The mean values of “perceived behavioral control”, “risk perception”, and “disaster experience” are higher in nonmodel communities than in model communities, which indicates that the public in nonmodel communities is more affected by the geological disaster environment, has higher risk perception, and has a higher evaluation of their ability to participate in disaster mitigation activities.

### 4.5. Binary Logistic Regression and Marginal Effects Results

Table 4 and Table 5 show the binary logistic regression results and marginal effect results of the factors influencing public participation in community disaster mitigation activities.

Holding other factors constant at the mean level, the evacuation drill activities revealed through the data in Table 4 and Table 5 that public participation in model communities is positively influenced by attitudes and perceived behavioral control factors (OR > 1), which can increase the likelihood of public participation by 5.7% and 6.1%, respectively. For nonmodel communities, the higher the public risk perception, the more severe the disaster experience, and the stronger the perception of participation is, the more they will increase the likelihood of participation by 6.9%, 9.9%, and 7.7%, respectively.

The behavioral decisions of residents in both types of communities in the self-help skills training activities were positively influenced by personal attitudes (OR > 1), which indicates that, regardless of the type of community, the public can be 7.4% more likely to participate by maintaining a positive attitude toward this activity compared with a negative attitude. The difference is that model communities are susceptible to the negative influence of two factors, perceived behavioral control and risk perception (OR < 1), which means that if these two factors show an increasing trend, it will lower the likelihood of public participation by 10.4% and 8.0%. Nonmodel communities’ perceived behavioral control and perception of group participation positively influence public participation behavior (OR > 1), indicating that the higher the level of perceived behavioral control and participatory cognition, the more likely it is to increase the level of public participation, with probabilities reaching 12.9% and 8.3%.

Attitude has a common positive influence (OR > 1) on opinion expression activities for both types of communities, increasing the likelihood of public participation by 17.3% and 14.2% for model and nonmodel communities. Increased levels of subjective norms and participatory cognition by the public in the model communities could increase their likelihood of participation by 5.2% and 6.6%, respectively. People in nonmodel communities were negatively influenced by subjective norms and disaster experiences, which reduced the likelihood of public participation by 7.6% and 6.5%, respectively, but they were positively influenced by perceived behavioral control, which indicated that it could promote public participation by 18.9%.

## 5. Discussion

### 5.1. Current Status of Public Participation in Disaster Mitigation Activities

According to the current state of public participation (Figure 3), the model disaster mitigation communities place a high value on training the public to escape and self-rescue abilities as part of the curriculum for daily disaster mitigation community development. We discovered that the Mulin community had more innovative management practices than other groups during our research and interviews. Using “grid-based” management, community residents were mobilized to participate in disaster risk reduction education activities, including studying disaster risk reduction brochures, organizing lectures to communicate knowledge, and practicing mudslide evacuation drills. Before the onset of the rainy season, the community conducts monthly large-scale evacuation drills, after which participants continue to learn first-aid methods such as stopping bleeding and dressing wounds. This also demonstrates the relatively well-developed emergency management system and disaster mitigation program of the model communities, which have strong community resilience and disaster preparedness advantages.

It is worth noting that the number of residents participating in “opinion expression” in model communities is less than in nonmodel communities. This implies, on the one hand, that model communities are organized and managed more comprehensively and meticulously and have a higher level of resident recognition of participation mechanisms, activity planning, implementation, and the total organization of mitigation activities. On the other hand, nonmodel communities still have shortcomings in the above aspects, and local people have a higher demand for participation and the active expression of feedback out of consideration for their own safety and the development of local disaster mitigation.

### 5.2. Influencing Factors on Public Participation in Disaster Mitigation Activities

(1) Factors influencing the “evacuation drill”

According to Table 4 and Table 5, residents in model communities are primarily influenced by attitudes and perceived behavioral control, whereas residents in nonmodel communities are influenced more by external natural environmental factors (risk perception and disaster experience) and social environment factors (perception of community participation) in evacuation drills. These contrasting sets of influences reflect the difference in the development of disaster mitigation communities: model communities have more complete resource allocation in the preparation of evacuation drills, such as evacuation signs, shelter directions, and warning evacuation brochures, etc., which can be seen everywhere and greatly meet the needs of residents while reducing the need for their various inputs. As a result, while choosing “evacuation drills,” inhabitants of the model communities were more likely to consider the value and social significance of the act itself, as well as their ability to conduct it, rather than the effect of the external environment. Nonmodel community residents were more likely to be motivated by the external environment, such as the threat of risk, the perception of the degree of local participation, and access to information, as well as their low monthly income, which makes their participation more passive if they continue to lack resources.

(2) Factors influencing “self-help skills training”

According to the findings, the more positive residents are toward self-help skills training activities, the more likely they are to support public participation. Therefore, regardless of the type of community, policymakers must emphasize the necessity of participation in their daily risk communication. Furthermore, this activity was unaffected by “subjective norms” and “disaster experience,” most likely because, as these skills are eminently in the public’s self-interest, the public is more concerned with acquiring self-rescue knowledge to safeguard their own interests.

In addition, an interesting result was found: the higher the perceived behavioral control and risk perception of the model community residents were, the less likely they were to participate in rescue skills training by approximately 10.4% and 8.0%, respectively, while other variables remained constant. This marginal effect result reflects the fact that overconfidence may lead to false competence assessment bias and that fear deters people from taking mitigating measures [75]. This also suggests that, with relatively saturated social resources and facility inputs, model disaster mitigation community construction needs to properly guide public perception to avoid generating excessive fear of disaster risks or overconfidence in one’s abilities. This also implies that there are serious challenges for policymakers to observe and channel extreme public emotions in community disaster prevention and mitigation construction.

Perceived behavioral control and participation cognition positively influenced public participation in nonmodel communities, demonstrating that the lower the “threshold” of public participation and the more knowledge regarding participation there is, the stronger the public’s incentive to engage. This indicates that there is still room for improvement in disaster mitigation community construction in nonmodel communities and that a large number of resources are still required to invest in and increase public participation in disaster mitigation, which is consistent with the actual situation of most rural disaster mitigation communities in China.

(3) Factors influencing the “opinion expression”

Consistent with Asare’s report [76], the factor of “attitude” was the most significant and important in influencing the feedback of community residents in both model and nonmodel communities (with 17.3% and 14.2% increases in willingness); i.e., people are more likely to be motivated to participate when they believe that their suggestions can contribute to the construction of community disaster mitigation.

The difference is that residents of model disaster mitigation communities are positively influenced by subjective norms and participatory cognition in expression activities, whereas residents of nonmodel disaster mitigation communities are negatively influenced by subjective norms and disaster experience and positively influenced by perceived behavioral control. This demonstrates that participation in model communities has a group effect [77], implying that it is important to develop a sense of “consensus” and provide transparent and convenient participation mechanisms for model communities to plan to mitigate disasters. In contrast, nonmodel communities are more independent in the decision-making process of participating in “opinion expression” activities, especially considering the influence of their experiences with disaster and the evaluation of their own capacity in the participation process [78,79]. With the participation status described in 5.1, it can be further illustrated that there is a greater possibility for public participation in nonmodel communities for disaster risk reduction. This is because residents of nonmodel communities take the independent initiative to participate in community disaster mitigation and do not rely on strategic plans from others. However, due to the lack of resource support and systematic arrangements for disaster preparedness and their own histories of tragedy in disasters, individual residents show higher demands and desires for participation (i.e., feedback) when they want to improve the current situation of community disaster mitigation construction.

### 5.3. Suggested Countermeasures for Building Disaster-Mitigating Communities

(1) Increasing the publicity of disaster mitigation and disaster mitigation activities to strengthen the awareness of residents to participate in risk reduction.

Table 5 demonstrates that attitude is a significant predictor of public engagement in the three types of disaster relief activities, indicating that residents are more likely to join when they perceive that their participation can offer more positive social meaning and value. As a result, successful recruitment to activities can be promoted by radio and mobile publicity vans. Furthermore, a collaborative network between schools and communities can create a mutually beneficial platform for both parties [80], including activities such as community science popularization by university students and risk identification and information lectures by professionals in disaster-prone areas.

(2) Reasonable allocation of community resources for disaster mitigation, strengthening the construction of disaster mitigation environments.

Based on the analysis of contributing factors, it is clear that community development in geological-disaster-prone locations must use resources wisely and concentrate on strengthening the disaster mitigation environment in nonmodel communities. For example, social resources should be encouraged to provide technical help and financial support, disaster mitigation education events should be arranged on a regular basis, and the “threshold” of participation should be lowered to allow more residents to participate. In daily disaster preparation, the most recent warnings and activities are distributed via WeChat groups, allowing residents to exchange comments. One-on-one, concentrated interventions are set up to help marginalized persons, particularly those who are severely impacted and have a strong subjective will. To minimize the limiting effects of overconfidence and pessimism, model disaster mitigation communities should focus on the observation and direction of the public’s attitude toward risk status and their ability to master rescue skills throughout the deployment of the “self-rescue skills training” plan.

(3) Carrying out “one leads many” community exchange activities and allowing model communities to play a leading role.

Communication across communities aids in identifying flaws and gaps within the communities themselves. Regular disaster mitigation communication efforts are carried out with the model community as the core and the neighboring rural communities as the targets of information. This provides a model for other communities to learn from in terms of setting up disaster mitigation activities and motivating people to participate. Organization factors as varied as the structure of the disaster mitigation working group, the development of emergency plans, and the design of disaster mitigation publicity, education, and training activities can all be improved by studying the successful experiences of the model communities.

## 6. Conclusions

Diversified community disaster mitigation activities encourage public participation and strengthen the community’s catastrophe prevention and mitigation and emergency response capacity through a two-way interaction process. However, the implementation of community disaster mitigation methods must take into account the current degree of community and public participation, as well as the elements that impact the public’s choice to participate in various activities. Therefore, this paper takes “evacuation drills”, “self-help skills training”, and “opinion expression” as examples of community-organized activities and compares model disaster mitigation communities with nonmodel communities to determine what specific factors influence the choice of public participation in disaster mitigation activities and the degree of influence under different levels of disaster mitigation infrastructural support.

The research results found that: (1) At present, there is a clear difference in the degree of public participation in disaster reduction activities between model disaster reduction communities and nonmodel communities, with model communities relying on resource support to have a stronger advantage in disaster reduction construction, while residents of nonmodel communities show a higher willingness and demand for participation. (2) Attitude is a key factor influencing public participation in disaster reduction activities, indicating that inducing positive public attitudes toward disaster mitigation activities is an important measure to increase the level of public participation across the board. (3) Community disaster reduction planning should reasonably allocate disaster reduction resources and improve the dynamic adjustment of phases. In the process of building future disaster reduction communities, model communities should make phased adjustments to their activity programs based on community characteristics and feedback from public participation and follow up on the public’s skill acquisition and subjective feelings promptly. The motivation of nonmodel communities’ residents to participate is influenced by the pathways to participation and the risk environment; therefore, decision-makers should focus on improving the resource allocation of nonmodel communities and enhancing the demonstration benefits of model communities.

There are still some limitations in this paper. First, the sample size only represents the specific situation of public participation in community disaster mitigation in Jinchuan County, a geohazard-prone area. The public community in other areas with different types or levels of risk may have different preferences affecting the results. Second, this paper explores specific factors that influence public participation but does not dig deeper into the interactions and path relationships among the factors; therefore, they can be further specified, extended, and supplemented in the future. The above issues need to be further discussed in future studies.

## Figures and Tables

**Table 1:** Demographic characteristics of the respondents.

Characteristic	Category	Model Communities		Nonmodel Communities	
		Frequency	Percentage	Frequency	Percentage
Gender	Male	44	39.29%	69	46.62%
	Female	68	60.71%	79	53.38%
Age	<18	0	0.00%	2	1.35%
	18–30	10	8.93%	17	11.49%
	31–45	13	11.61%	36	24.32%
	46–60	43	38.39%	64	43.24%
	60–80	46	41.07%	29	19.59%
Educational level	Primary school or below	20	17.86%	56	37.84%
	Junior high school	34	30.36%	46	31.08%
	Senior high/vocational high school	21	18.75%	17	11.49%
	Technical school	22	19.64%	19	12.84%
	Undergraduate degree or above	15	13.39%	10	6.76%
Occupation	Student	5	4.46%	5	3.38%
	Farmer	34	30.36%	103	69.59%
	Civil servant	10	8.93%	13	8.78%
	Surveyor	10	8.93%	6	4.05%
	Staff	2	1.79%	6	4.05%
	Teacher	8	7.14%	6	4.05%
	Self-employed	34	30.36%	8	5.41%
	Retirement	9	8.04%	1	0.68%
Monthly income	<500 RMB	26	23.21%	60	40.54%
	500–1500 RMB	16	14.29%	37	25.00%
	1500–3000 RMB	17	15.18%	22	14.86%
	3000–4000 RMB	16	14.29%	11	7.43%
	>4000 RMB	37	33.04%	18	12.16%

**Table 2:** Rotated component matrix.

	Independent Variable					
	Attitudes	Subjective Norm	Perceived Behavioral Control	Risk Perception	Disaster Experience	Participatory Cognition
Q11	0.824	0.128	0.088	0.157	−0.082	0.162
Q12	0.617	0.386	0.067	−0.133	0.026	0.094
Q13	0.822	0.200	0.066	0.115	0.068	0.144
Q21	0.321	0.642	0.125	0.006	−0.029	0.099
Q22	0.095	0.851	0.092	−0.017	0.014	0.149
Q23	0.196	0.806	0.075	−0.032	−0.028	0.129
Q24	0.094	0.789	0.025	0.032	−0.021	0.152
Q31	0.177	0.002	0.786	0.048	0.034	−0.020
Q32	−0.023	0.103	0.745	0.025	−0.011	0.129
Q33	0.017	0.038	0.807	−0.020	−0.066	0.204
Q34	0.064	0.145	0.820	−0.011	−0.048	−0.023
Q41	0.021	0.003	0.001	0.826	0.126	−0.034
Q42	−0.036	0.021	0.062	0.820	0.080	−0.007
Q43	0.231	−0.056	−0.027	0.767	0.125	0.033
Q44	0.101	−0.012	−0.012	0.750	0.213	−0.028
Q51	0.001	−0.003	0.042	0.153	0.863	−0.021
Q52	0.001	−0.024	−0.059	0.073	0.835	0.100
Q53	−0.007	−0.010	−0.093	0.248	0.746	0.037
Q61	0.103	0.102	0.028	0.003	0.051	0.793
Q62	0.188	0.209	0.102	−0.037	0.049	0.783
Q63	0.100	0.160	0.100	−0.002	0.019	0.833

**Table 3:** Levels of the variability of impact factors between different community types.

	Community Type	N	M	S.D.	t	df	Sig.
Attitude	Model Communities	112	4.740	0.498	2.737	258	0.007
	Nonmodel Communities	148	4.530	0.674			
Subjective norm	Model Communities	112	4.768	0.433	2.213	258	0.028
	Nonmodel Communities	148	4.635	0.510			
Perceived behavioral control	Model Communities	112	4.120	1.097	−2.731	258	0.007
	Nonmodel Communities	148	4.450	0.851			
Risk perception	Model Communities	112	3.815	0.857	−4.079	258	0.000
	Nonmodel Communities	148	4.228	0.771			
Disaster experience	Model Communities	112	1.790	0.902	−2.017	258	0.045
	Nonmodel Communities	148	2.030	0.979			
Participatory cognition	Model Communities	112	4.355	0.489	2.757	258	0.006
	Nonmodel Communities	148	4.184	0.499			

**Table 4:** Binary logistic model regression results.

Variable	Evacuation Drills (Odds Ratio)		Self-Rescue Skills Training (Odds Ratio)		Opinion Expression (Odds Ratio)	
	Model Communities	Nonmodel Communities	Model Communities	Nonmodel Communities	Model Communities	Nonmodel Communities
Gender	0.430 *	0.584	3.015 ***	1.550	1.481	0.449 *
Age	0.916 ***	0.951 **	0.999	1.030	1.047	0.990
Education level	0.980	1.886 *	1.166	0.742	4.006 ***	1.242
Career	1.031	1.127	1.166	1.260	1.636 *	1.459 **
Attitude	1.393 *	1.141	1.415 *	1.732 *	15.786 **	2.235 ***
Subjective norm	0.918	0.862	0.866	0.821	2.293 *	0.649 *
Perceptual behavior control	1.423 *	0.938	0.612 **	2.622 *	0.947	2.928 ***
Risk perception	0.889	1.661 **	0.686 *	1.451	1.136	1.231
Disaster experience	0.867	2.060 ***	1.040	0.872	1.585	0.691 *
Participatory cognition	1.243	1.759 **	1.058	1.852 **	2.888 *	1.258
_cons	19.776	159.435 **	0.056	0.001 **	0.000 **	2.716
McKelvey and Zavoina’s R2	0.224	0.247	0.109	0.211	0.361	0.241
Log-likelihood	−76.895	−62.703	−90.438	−60.990	−31.026	−77.342

Note: * *p* < 0.1, ** *p* < 0.05, *** *p* < 0.01.

**Table 5:** Binary logistic regression marginal effect results.

Variable	Evacuation Drills (dy/dx)		Self-Rescue Skills Training (dy/dx)		Opinion Expression (dy/dx)	
	Model Communities	Nonmodel Communities	Model Communities	Nonmodel Communities	Model Communities	Nonmodel Communities
Gender	−0.146 **		0.234 ***			−0.141 **
Age	−0.015 ***	−0.007 **				
Education level		0.087 **			0.087 ***	
Career					0.031 *	0.066 **
Attitude	0.057 *		0.074 *	0.074 *	0.173 **	0.142 ***
Subjective norm					0.052 *	−0.076 **
Perceptual behavior control	0.061 *		−0.104 **	0.129 *		0.189 ***
Risk perception		0.069 *	−0.080 *			
Disaster experience		0.099 ***				−0.065 *
Participatory cognition		0.077 **		0.083 **	0.066 **	

Note: * *p* < 0.1, ** *p* < 0.05, *** *p* < 0.01.
